# The International Congress of Hygiene and Demography

**Published:** 1894-06

**Authors:** 


### The International Congress of Hygiene and Demography.

A British Committee, of which Sir Douglas Galton is the Chairman, and Professor W. H. Corfield is the Treasurer, has been formed to further the interests in this country of the Eighth International Congress of Hygiene and Demography, which is to be held in Budapest, from the 1st to the 8th of September this year. Any information may be obtained about the Congress from the Hon. Secretary, Dr. Paul F. Moline, 42 Walton Street, London, S.W.]

